# Supplementary material for: Heterogeneous trajectories of exercise self-efficacy and its predictors in patients with multivessel coronary artery disease: A longitudinal study
Source: PLoS One. 2026 Feb 27;21(2):e0339591. doi: 10.1371/journal.pone.0339591 (PMC12948052; doi:10.1371/journal.pone.0339591)
Supplement: S1 File — (DOCX) [file pone.0339591.s001.docx]

**S2 Table. Analysis of systematic differences between included and excluded patients with multivessel coronary artery disease (*n* = 371)**

| Variables | Included group | Excluded group | *χ^2^/t* | *P* |
| --- | --- | --- | --- | --- |
| Gender, *n* (%) |  |  | 1.602 | 0.206 |
| Male | 198(66.7) | 55(74.3) |  |  |
| Female | 99(33.3) | 19(25.7) |  |  |
| Age (years), *n* (%) |  |  | 0.016 | 0.898 |
| ＜60 | 102(34.3) | 26(35.1) |  |  |
| ≥60 | 195(65.7) | 48(64.9) |  |  |
| Educational level (years), *n* (%) |  |  | 1.391 | 0.499 |
| Elementary (≤6) | 80(26.9) | 15(20.3) |  |  |
| Secondary (7-9) | 142(47.8) | 39(52.7) |  |  |
| Higher (>9) | 75(25.3) | 20(27.0) |  |  |
| Marital status, *n* (%) |  |  | 0.208 | 0.648 |
| Single | 16(5.4) | 5(6.8) |  |  |
| Married | 281(94.6) | 69(93.2) |  |  |
| Residential type, *n* (%) |  |  | 1.338 | 0.247 |
| Living alone | 14(4.7) | 6(8.1) |  |  |
| Living with others | 283(95.3) | 68(91.9) |  |  |
| Employment status, *n* (%) |  |  | 3.032 | 0.082 |
| Employed | 154(51.9) | 30(40.5) |  |  |
| Unemployed | 143(48.1) | 44(59.5) |  |  |
| Residential location, *n* (%) |  |  | 0.086 | 0.769 |
| Rural | 126(42.4) | 30(40.5) |  |  |
| Urban | 171(57.6) | 44(59.5) |  |  |
| Average monthly household income (RMB), *n* (%) |  |  | 1.671 | 0.434 |
| ＜¥3000 | 104(35.0) | 21(28.4) |  |  |
| ¥3000~5000 | 110(37.0) | 33(44.6) |  |  |
| ＞¥5000 | 83(28.0) | 20(27.0) |  |  |
| Exercise habit, *n* (%) |  |  | 0.579 | 0.447 |
| Yes | 135(45.5) | 30(40.5) |  |  |
| No | 162(54.5) | 44(59.5) |  |  |
| Treatment method, *n* (%) |  |  | 0.460 | 0.498 |
| Medication alone | 67(22.6) | 14(18.9) |  |  |
| PCI or CABG | 230(77.4) | 60(81.1) |  |  |
| Family history of coronary heart disease, *n* (%) |  |  | 0.735 | 0.391 |
| Yes | 144(48.5) | 40(54.1) |  |  |
| No | 153(51.5) | 34(45.9) |  |  |
| Hypertension, *n* (%) |  |  | 0.329 | 0.566 |
| Yes | 186(62.6) | 49(66.2) |  |  |
| No | 111(37.4) | 25(33.8) |  |  |
| Diabetes, *n* (%) |  |  | 0.899 | 0.343 |
| Yes | 99(33.3) | 29(39.2) |  |  |
| No | 198(66.7) | 45(60.8) |  |  |
| Hyperlipidemia, *n* (%) |  |  | 1.089 | 0.297 |
| Yes | 18(6.1) | 7(9.5) |  |  |
| No | 279(93.9) | 67(90.5) |  |  |
| Stroke, *n* (%) |  |  | 3.608 | 0.057 |
| Yes | 23(7.7) | 11(14.9) |  |  |
| No | 274(92.3) | 63(85.1) |  |  |
| Exercise self-efficacy score, mean ± SD | 33.85±11.30 | 32.00±12.53 | 1.233 | 0.218 |
| Social support score, mean ± SD | 14.49±4.05 | 13.77±3.77 | 1.394 | 0.164 |
| Anxiety score, mean ± SD | 10.00±4.64 | 10.22±4.59 | -0.370 | 0.711 |

Note: CABG, Coronary Artery Bypass Grafting; PCI, Percutaneous Coronary Intervention.
