# Supplementary material for: Heterogeneous trajectories of exercise self-efficacy and its predictors in patients with multivessel coronary artery disease: A longitudinal study
Source: PLoS One. 2026 Feb 27;21(2):e0339591. doi: 10.1371/journal.pone.0339591 (PMC12948052; doi:10.1371/journal.pone.0339591)
Supplement: S2 Table — (DOCX) [file pone.0339591.s002.docx]

**S3 Table. Results of 10-Fold Cross-Validation for Sensitivity Analysis**

| Metric | Model Type | Training  Set (Mean ± SD) | Validation  Set (Mean ± SD) | Interpretation |
| --- | --- | --- | --- | --- |
| Entropy (Classification Accuracy) | 3-class Model | 0.918 ± 0.012 | 0.905 ± 0.015 | Higher entropy indicates more reliable subgroup classification. |
|  | 4-class Model | 0.902 ± 0.014 | 0.887 ± 0.018 | Lower entropy than 3-class model, reflecting less accurate classification. |
| VLMRT (P-value) | 3-class Model | < 0.001 | < 0.001 | Highly significant, supporting strong fit improvement of the 3-class model. |
|  | 4-class Model | 0.008 ± 0.002 | 0.012 ± 0.003 | Weaker significance than 3-class model, indicating less support for 4-class fit. |
| Subgroup Proportion Consistency | 3-class Model | Proportion difference < 5% | Proportion difference < 5% | Consistent subgroup patterns across sets, ensuring clinical interpretability. |
|  | 4-class Model | Proportion fluctuation > 10% | Proportion fluctuation > 10% | Unstable fourth subgroup, with smallest proportion < 15% (limited clinical use). |

Note: SD = Standard deviation; VLMR T = Vuong-Lo-Mendell-Rubin Test. The analysis was repeated 10 times, and mean values ± SD were reported for continuous metrics.
