# Supplementary material for: Heterogeneous trajectories of exercise self-efficacy and its predictors in patients with multivessel coronary artery disease: A longitudinal study
Source: PLoS One. 2026 Feb 27;21(2):e0339591. doi: 10.1371/journal.pone.0339591 (PMC12948052; doi:10.1371/journal.pone.0339591)
Supplement: Attachment — Submitted filename: Response to Reviewers.docx [file pone.0339591.s007.docx]

October 23, 2025

Hossein Ali Adineh, Ph.D

Academic Editor

*PLOS ONE*

Dear Dr. Hossein Ali Adineh,

I am writing on behalf of my co-authors to submit our revised manuscript PONE-D-25-35625, entitled “Heterogeneous trajectories of exercise self-efficacy and its predictors in patients with multivessel coronary artery disease: a longitudinal study” to be considered for publication in *PLOS ONE*.

We wish to thank you and the two reviewers for your thoughtful comments and suggestions. In this revised version, we have made revisions according to your requirements and the comments from two reviewers. Point-by-point responses to the editor and two reviewers are listed below this letter. To facilitate this discussion, we retyped your comments in *italic font* and then present our responses.

All authors have read, and approved submission of the manuscript and this manuscript has not been published and is not being considered for publication elsewhere in whole or part in any language. All authors meet the 4 ICMJE criteria for authorship.

Thank you for reviewing and providing your suggestions on improving our manuscript. I am available to address any questions that might arise during the resubmission process. We appreciate the time and effort that you and the editorial reviewers have devoted to considering our manuscript.

Sincerely,

Jianhui Wang, PhD, RN

Nurse Administration Department, Tangshan Gongren Hospital, Tangshan, China.

E-mail: anita30@163.com

PONE-D-25-35625

**Authors’ responses to the editor**

**Comment 1:**

*Please ensure that your manuscript meets PLOS ONE's style requirements, including those for file naming.*

*https://journals.plos.org/plosone/s/file?id=wjVg/PLOSOne_formatting_sample_main_body.pdf and*

*https://journals.plos.org/plosone/s/file?id=ba62/PLOSOne_formatting_sample_title_authors_affiliations.pdf.*

**Response 1:**

We sincerely thank the editor for your careful review. As suggested, we have revised the format of the full text and the file naming to meet the style requirements of *PLOS ONE*. Thank you again for your helpful suggestions.

**Comment 2:**

*We note that the grant information you provided in the ‘Funding Information’ and ‘Financial Disclosure’ sections do not match.*

*When you resubmit, please ensure that you provide the correct grant numbers for the awards you received for your study in the ‘Funding Information’ section.*

**Response 2:**

We apologize for this oversight. In our resubmitted cover letter, we provided the correct funding information, which is “Funding Information

This study was supported by the Medical Science Research Project of Hebei (No.20241512).”

Thank you again for your careful review.

**Comment 3:**

*We note that your Data Availability Statement is currently as follows: [All relevant data are within the manuscript and its Supporting Information files.]*

*Please confirm at this time whether or not your submission contains all raw data required to replicate the results of your study. Authors must share the “minimal data set” for their submission. PLOS defines the minimal data set to consist of the data required to replicate all study findings reported in the article, as well as related metadata and methods (https://journals.plos.org/plosone/s/data-availability#loc-minimal-data-set-definition).*

*For example, authors should submit the following data:*

*- The values behind the means, standard deviations and other measures reported;*

*- The values used to build graphs;*

*- The points extracted from images for analysis.*

*Authors do not need to submit their entire data set if only a portion of the data was used in the reported study.*

**Response 3:**

Thank you very much for your detailed review and valuable comments. All raw data underlying figures and analyses are provided in Supporting Information (S4 Data).

**Comment 4:**

*Thank you for stating the following in the Acknowledgments Section of your manuscript:*

*“This study was supported by the Medical Science Research Project of Hebei (No.20241512).”*

*We note that you have provided additional information within the Acknowledgements Section that is not currently declared in your Funding Statement. Please note that funding information should not appear in the Acknowledgments section or other areas of your manuscript. We will only publish funding information present in the Funding Statement section of the online submission form.*

*Please remove any funding-related text from the manuscript and let us know how you would like to update your Funding Statement. Currently, your Funding Statement reads as follows:*

*“The author(s) received no specific funding for this work.”*

*Please include your amended statements within your cover letter; we will change the online submission form on your behalf.*

**Response 4:**

Thank you for your careful review. We have removed the descriptions of the funding information in the Acknowledgements Section and manuscripts. According to your suggestion, we have attached the revised statement to the cover letter. Thank you once again for your helpful comments.

**Comment 5:**

*Please include a caption for figure 1.*

**Response 5:**

Page 10, line 227-231:

Thank you for your detailed review. Based on your suggestion, we have added a caption for Figure 1, which is “**Fig 1. Three latent classes of exercise self-efficacy trajectories in patients with multivessel coronary artery disease.** This figure illustrates the development of exercise self-efficacy over four time points (T1 to T4) across three latent classes: Class 1 (22%, Low-Efficacy Decline Group), Class 2 (34%, High-Efficacy Ascending-Stable Group), and Class 3 (44%, Moderate-Efficacy Continuous Increase Group). Error bars represent standard errors.” And we have modified the format and naming of the figure according to your requirements. Thank you once again for your careful consideration.

**Comment 6:**

*If the reviewer comments include a recommendation to cite specific previously published works, please review and evaluate these publications to determine whether they are relevant and should be cited. There is no requirement to cite these works unless the editor has indicated otherwise.*

**Response 6:**

Thank you for your valuable comment. We have carefully reviewed all reviewer comments and checked for any recommendations to cite specific previously published works. Since there were no such specific citations suggested by the reviewers, we have confirmed that all included references are relevant to our study’s content, methodology, and discussions, and we have not added any citations that are not necessary or indicated by the editor.

PONE-D-25-35625

**Authors’ responses to the reviewers**

(Note: Due to major revision of the manuscript, pages noted by reviewer which refer to the original manuscript differ that those identified in authors responses which refer to the revised manuscript.)

**Replies to Reviewer #1:**

**Major concerns:**

**Comment 1:** *The study population was patients with multivessel coronary artery disease. However, within this population, it is possible that there is a wide range of clinical severity of disease that could confound findings. Did authors consider how clinical severity of coronary artery disease may affect results?*

**Response 1:**

We sincerely appreciate the reviewer’s insightful comment regarding the potential confounding effect of clinical severity of disease within the multivessel coronary artery disease population.

We acknowledge that the lack of detailed assessments of clinical severity (e.g., Gensini score, extent of stenosis in each diseased vessel) is a limitation of our current study. To minimize the potential impact of variability in clinical severity as much as possible, we strictly defined the inclusion criteria for “multivessel coronary artery disease” as “≥ 2 major coronary arteries with ≥ 50% luminal stenosis confirmed by coronary angiography” to ensure a relatively homogeneous group in terms of baseline disease extent (Page 4, line 83).

Recognizing this gap, in future research, we plan to incorporate comprehensive measures of clinical severity (e.g., Gensini scoring, detailed angiographic findings) to further investigate the interplay between disease severity and exercise self-efficacy trajectories in this population.

Page 22, line 446 to Page 23, line 450:

According to your suggestion, we have added the following content to the Limitations section of the manuscript, which is “Third, the study did not include detailed assessments of clinical severity of multivessel coronary artery disease, such as the Gensini score or the extent of stenosis in each diseased vessel. This may restrict a comprehensive evaluation of disease severity and its potential influence on the results. Future studies could include multiple indicators to assess disease severity more thoroughly, such as the Gensini score and left ventricular ejection fraction, to further validate our findings.”

Thank you again for helping us strengthen the manuscript.

**Minor concerns:**

**Comment 1:**

*Abstract – consider briefly defining developmental trajectory of exercise self-efficacy as many readers of PLOS one will be unfamiliar with this terminology.*

**Response 1:**

Page 1, line 4-5:

Thank you for your constructive comments. Based on your suggestions, we have added a brief definition to the development trajectory of exercise self-efficacy to help readers better understand, and to read “The developmental trajectory of exercise self-efficacy refers to the course of change in an individual's belief in their capability to successfully perform exercise-related behaviors over time.” We sincerely thank you for your detailed suggestions from the reader’s point of view. Thank you again for your helpful comments.

**Comment 2:**

*Line 33-34. The line starting with “despite evidence that…” is an incomplete sentence and I believe should be combined with the following one or revised.*

**Response 2:**

Page 2, line 35-37:

Thank you so much for your careful review. According to your comments, we have modified the sentence beginning with “despite evidence that...” to merge with the following sentence, to read “Despite evidence that exercise rehabilitation improves cardiovascular function, exercise capacity, and quality of life [5,6], existing studies indicate that the current state of exercise rehabilitation across various countries is concerning.” Thank you again for your detailed comments.

**Comment 3:**

*Line 38: How is optimal level defined?*

**Response 3:**

Page 2, line 40-41:

We sincerely appreciate the reviewer's valuable comment, which contributes to enhancing the clarity and precision of our manuscript. Regarding the definition of “optimal level” in the original line 38, our intended meaning is that the patients' participation rate is far below what is recommended by cardiac rehabilitation guidelines. To make this clearer, we have revised the relevant content in the manuscript. The original sentence has been modified to “far below the minimum level recommended by cardiac rehabilitation guidelines for effective cardiac recovery” to explicitly indicate that it is the minimum requirement from the guidelines that is not met. Thank you again for your constructed comments.

**Comment 4:**

*Line 85-89: Not sure how relevant your sample size calculations are, consider removing*

**Response 4:**

Page 4, line 92 to Page 5, line 103:

Thank you for your rigorous comments. Regarding the concern about the relevance of the sample size calculations and the suggestion of removing this section, we would like to emphasize that sample size calculation is a crucial component of study design to ensure the statistical validity and reliability of the results. In view of your consideration, we found that the Sample size calculation in the original writing of “2.3. Sample size” did not highlight the correlation with this study. Therefore, in response to the reviewer's doubts about the lack of direct relevance in the study sample size calculation, we have revised the corresponding section in the manuscript to better contextualize the sample size calculation within the overall aims of our study, thereby enhancing the clarity of its importance without removing this key methodological component, which is “The sample size determination was conducted using G*power 3.1 software. Given that our study involved tracking exercise self-efficacy trajectories derived from four repeated measurements, we employed the Single-group repeated-measures analysis of variance algorithm [20]. The nonsphericity correction (ε) was set to 0.5, a value widely utilized in repeated-measures designs when the precise correlation structure between repeated measurements is not fully established in advance. This value accounts for a moderate deviation from sphericity, thus ensuring the analysis remains robust across diverse potential correlation patterns among the four measurement points. The statistical power was set to 0.8, which strikes a balance between the risk of Type II errors and the practicality of recruiting participants, guaranteeing the study a reasonable likelihood of identifying meaningful trajectory patterns if they exist. With 95% confidence intervals (CI), these parameters resulted in a minimum required sample size of 182. Considering a potential 20% attrition rate, a total of 219 participants were deemed necessary to ensure sufficient statistical power for analyzing the exercise self-efficacy trajectories across the four measurement points.” We believe this revision will make the relevance of the sample size calculations more apparent and strengthen the methodological rigor of our study. Thank you again for your careful consideration.

**Comment 5:**

*Line 94. Please define exercise habit. This characteristic ends up being an important factor for exercise self-efficacy so a clear understanding of how this is defined both in the methods and in context of your discussion is crucial.*

**Response 5:**

Page 5, line 114-118:

Thank you for your excellent suggestions. Based on your comments, we have revised the definition of exercise habit, which is “In this study, we defined the participants as having an exercise habit if they engaged in physical activity for a cumulative duration of at least 30 minutes per day on at least three days per week within the three months prior to hospitalization, including aerobic exercises such as walking, jogging, swimming, and cycling, as well as anaerobic exercises mainly consisting of low-load resistance training.”

Page 20, line 383-388:

Additionally, we have also ensured that the discussion of exercise habit in the Discussion section is consistent with this definition, so as to provide a coherent narrative regarding its role in relation to exercise self-efficacy. Based on your comments, we have added: “Exercise habits in our study reflect a sustained and diversified pattern of physical activity. Aerobic exercises, with their focus on cardiovascular endurance, lay the foundation for sustaining physical activity, while low-load resistance training enhances muscular strength and endurance, which are essential for performing various physical tasks with confidence. Such an exercise habit ensures that patients not only improve their cardiovascular function but also develop a sense of mastery over their physical capabilities through diverse exercise experiences, which is a core component of exercise self-efficacy. ” Thank you once again for your careful consideration.

**Comment 6:**

*Line 95: Were any other comorbidities in addition to hypertension and diabetes considered? Why only include these?*

**Response 6:**

Thank you for your detailed comment. To address this, we would like to clarify that we did consider other comorbidities, including hyperlipidemia and stroke, and collected relevant data. We focused on hypertension and diabetes due to their high prevalence in multivessel coronary artery disease patients (hypertension: 63.34%, diabetes: 34.50% in our study) and their well-documented roles as independent risk factors for cardiovascular outcomes and exercise self-efficacy. Hyperlipidemia and stroke were also evaluated but not prioritized for analysis, as their relatively lower prevalence (hyperlipidemia: 6.73%, stroke: 9.16% in our study) limited the statistical power to detect meaningful associations with exercise self-efficacy trajectories (S2 Table and S4 Data). This selection ensured our analysis concentrated on factors most relevant to our study’s objectives while acknowledging the consideration of other comorbidities.

To address any confusion, we have included in Table 4 the analysis of patients with multivessel coronary artery disease with hyperlipidemia and stroke. The results showed that there was no significant difference in hyperlipidemia (*χ^2^* = 1.796, *P* = 0.407) and stroke (*χ^2^* = 2.968, *P* = 0.227) among the three trajectory subgroups of exercise self-efficacy in patients with multivessel coronary artery disease (*P* > 0.05). This result had no effect on the results of the Multinomial logistics regression analysis in Table 5.

Page 5, line 113-114:

Based on your suggestions, we have added the corresponding description in the “2.4.1. Demographic and clinical characteristics” section of the “2.4. Measures”, which is “hyperlipidemia, and stroke”.

Page 9, line 199:

In addition, we have added the corresponding description in the “3.1. Characteristics of the participants” section of the “3. Results”, which is “6.1% had hyperlipidemia, and 7.7% had stroke.”

**Comment 7:**

*Line 101: Consider providing a brief description of the three dimensions (task efficacy, coping efficacy, and scheduling efficacy) to allow readers to better understand the main measurement outcome of your study.*

**Response 7:**

Page 6, line 124-131:

Thank you for your valuable suggestions. Based on your comments, we have added a brief description of the three dimensions (task efficacy, coping efficacy, and scheduling efficacy), which is “Task efficacy refers to an individual’s confidence in mastering the elemental aspects of exercise (e.g., using proper exercise techniques, following instructor directions, and performing all required movements); coping efficacy denotes an individual’s confidence in sustaining exercise participation under challenging circumstances (e.g., exercising when feeling discomfort, lacking energy, or unwell); and scheduling efficacy represents an individual’s confidence in integrating exercise into daily life despite time constraints (e.g., including exercise in daily routines, consistently exercising at least three times a week, and arranging schedules to ensure regular exercise).” Thank you again for your excellent suggestions.

**Comment 8:**

*Line 117: What was the training and qualifications required for researchers?*

**Response 8:**

Page 7, line 147-150:

Thank you for your careful review. Based on your comments, we have specified the training and qualifications of researchers, which is “Specifically, the researchers were master’s degree candidates in nursing, who met the required qualifications by completing specialized training in clinical research methods. This training covered ethical principles in human subject research, standardized data collection procedures, and effective patient communication—all essential for conducting clinical investigations involving patient populations.” Thank you again for your detailed comments.

**Comment 9:**

*Line 119: By investigation here, do you mean specifically survey administration?*

**Response 9:**

Yes, by “investigation” here, we specifically mean survey administration. We used standardized questionnaires to collect data from the participants, which involved distributing, guiding participants to complete, and retrieving the surveys.

Page 7, line 151-152:

To avoid the ambiguity caused by the word “investigation”, we have revised this sentence, to read “Before the survey, the purpose, significance, and confidentiality measures were explained to the participants, and the survey proceeded only after obtaining their informed consent.” Thank you again for your valuable comments.

**Comment 10:**

*Line 129: Was the previous longitudinal study looking specifically and exercise efficacy?*

**Response 10:**

Page 7, line 164-166:

Thank you for your careful review. The “previous longitudinal study” was not specifically focused on exercise efficacy. Based on your comments, we have revised the relevant content in the manuscript to clarify this point, which is “A previous study, which focused on behavior patterns and physical function in stroke patients, found that the time points for longitudinal investigations of patients were predominantly established during hospitalization, as well as at one month, three months, and six months after discharge.” Thank you again for your helpful suggestions.

**Comment 11:**

*Table 4 &5: For average monthly income, is the middle category 3000-5000?*

**Response 11:**

Table 4 &5:

Yes. Thank you for your detailed review. Based on your proposal, we have revised the middle category of average monthly income to ¥3000-5000. Thank you again for your excellent suggestion.

**Comment 12:**

*Table 4: For the social support and anxiety rows, please clarify that these are the mean scores from the questionnaires.*

**Response 12:**

Table 4

Thank you for your careful consideration. We have clarified in Table 4 that the values for “Social support” and “Anxiety” represent the mean scores derived from the respective questionnaires. Specifically, we revised the row labels to “Social support score (mean ± SD)” and “Anxiety score (mean ± SD)” to make this explicit. Thank you again for your helpful comment.

**Replies to Reviewer #2:**

**Comment 1:**

*Measurement timing: Do the selected follow-up points (day 3, 1, 3, 6 months) adequately capture changes in exercise self-efficacy, or would a longer follow-up (e.g., 12 months) provide a clearer trajectory?*

**Response 1:**

Thank you for your insightful comment regarding the measurement timing of follow-up points.

The selected follow-up points (day 3, 1, 3, 6 months) were determined based on the conventional design in previous studies, consensus, and the transtheoretical model. These indicate that notable changes in exercise self-efficacy can be observed within the first 6 months following intervention or hospital discharge. These time points enable us to capture the short- to medium-term dynamics of exercise self-efficacy.

However, we fully agree with your suggestion that a longer follow-up (e.g., 12 months) could potentially offer a clearer long-term trajectory of exercise self-efficacy. Exercise self-efficacy is influenced by various factors over time, and a 12-month follow-up would allow for the observation of more prolonged changes and patterns.

Therefore, based on your suggestions, we have made the following changes in the Methods, Discussion, and Limitations section.

Page 7, line 166 to Page 18, line 169:

In the Methods section, we revised the original text to “Consequently, we selected follow-up periods of one month (T2), three months (T3), and six months (T4) after discharge to explore the short- to medium-term changes in exercise self-efficacy in patients with multivessel coronary artery disease”, which clarifies that our study investigates the short- to medium-term changes in exercise self-efficacy among patients with multivessel coronary artery disease.

Page 15, line 265-270:

In addition, we have added the following content in the Discussion section, to read: “While the follow-up points in this study provide insights into the short- to medium-term changes in exercise self-efficacy, the recommendation for a longer follow-up is pertinent. A longer follow-up would facilitate a more thorough understanding of the long-term trajectory of exercise self-efficacy, uncovering potential sustained changes or evolving patterns over an extended duration. Future research with longer follow-up periods is needed to further explore the long-term dynamics of exercise self-efficacy in patients with multivessel coronary artery disease.”

Page 22, line 443-446:

Furthermore, we restated this point in the Limitations section, to read “Second, the follow-up period was limited to six months, preventing an exploration of the long-term trajectory of exercise self-efficacy. Future studies should extend the follow-up period to obtain a more comprehensive understanding of exercise self-efficacy trajectories in patients with multivessel coronary artery disease.”

Thank you again for your thoughtful suggestion.

**Comment 2:**

*Generalizability: Recruitment from three tertiary hospitals in Tangshan may limit external validity. How might regional and healthcare system factors affect the findings?*

**Response 2:**

Thank you for your valuable comments. We acknowledge that recruiting participants exclusively from three tertiary hospitals in Tangshan may limit the generalizability of our findings to other populations or settings. Despite these limitations, our sample is representative of cardiac rehabilitation patients in tertiary hospitals within similar socioeconomic contexts, and the findings retain value for guiding clinical practice in comparable settings. To strengthen generalizability, we suggest future studies include participants from diverse healthcare settings (e.g., primary care, community hospitals) and geographic regions.

Page 22, line 439-443:

This is a valid limitation, and we have restated this point in the Limitations section, to read “First, the study subjects were drawn exclusively from three tertiary hospitals in Tangshan, which may limit the regional representativeness of the sample and affect the generalizability of the results. Future studies could expand the sample size to include patients from diverse regions and varying medical conditions to enhance the applicability of the findings.”

Thank you again for your helpful suggestion.

**Comment 3:**

*Attrition and missing data: How were exclusions and loss to follow-up handled? Were there systematic differences between included and excluded patients that could bias results?*

**Response 3:**

Thank you for your careful consideration. According to your suggestion, we have addressed these issues as follows:

Regarding handling of exclusions and loss to follow-up:

Attrition was defined as participants who failed to complete any scheduled follow-up assessment and could not be contacted after three attempts (via phone, hospital records, and family contact, each attempt spaced 72 hours apart). A total of 371 patients were initially enrolled, with 74 lost to follow-up, resulting in 297 patients who completed all four follow-up assessments. For these 297 patients, no missing data were present across all variables (including exercise self-efficacy and demographic characteristics), so we did not employ methods for missing values.

Analysis of systematic differences between included and excluded patients:

To assess potential bias, we compared key baseline characteristics (gender, age, education level, marital status, residential type, employment status, residential location, average monthly household income, exercise habit, treatment method, family history of coronary artery disease, hypertension, diabetes, hyperlipidemia, stroke,and baseline exercise self-efficacy, social support, anxiety) between included (297 patients) and excluded (74 patients) groups using chi-square tests (for categorical variables) and independent-samples t-tests (for continuous variables). No statistically significant differences were observed between the two groups (all	*P* > 0.05), indicating that exclusions and loss to follow-up did not introduce systematic bias into our results.

Based on your comments, we have supplemented a new table (S2 Table , Supplementary Material) summarizing the baseline characteristics of included vs. excluded patients has been added to provide direct visual evidence of no significant group differences.

Page 8, line 191 to Page 9, line 193:

In addition, we have added a description of this result in “3.1. Characteristics of the participants” in the results section, to read “To address potential bias from attrition, we compared baseline characteristics between included and excluded patients with multivessel coronary artery disease, and no statistically significant differences were observed (all *P* > 0.05; S2 Table).”

Thank you again for your constructive comments.

**Comment 4:**

*Trajectory model choice: Both 3-class and 4-class models showed fit. Why was the 3-class solution preferred? A sensitivity check comparing solutions could add confidence.*

**Response 4:**

Thank you very much for your valuable comments. We chose the 3-class solution for the following reasons:

a. Model fit indices: While both the 3-class and 4-class models demonstrated acceptable fit, the 3-class model showed more favorable performance across key indices. For instance, the aBIC (Sample-Size Adjusted BIC) for the 3-class model (8194.664) was lower than that of the 4-class model (8097.768) — lower aBIC values indicate better model fit, especially when accounting for sample size. Additionally, the Entropy (0.922 for 3-class vs. 0.911 for 4-class) was higher for the 3-class model, suggesting greater classification accuracy of individuals into latent classes.
b. Substantive interpretability: The 3-class model yielded latent trajectory groups with clear, meaningful, and distinct patterns of exercise self-efficacy change over time. In contrast, the 4-class model produced a smaller class (with a relatively low proportion, as seen in the “Latent trajectory” column: 0.17 for one of the classes) that was difficult to interpret substantively and did not add unique theoretical value to understanding exercise self-efficacy trajectories.

To further address your concern about confidence in model selection, we conducted a 10-fold cross-validation as the sensitivity analysis, which is a widely recognized method to assess model stability in latent class growth modeling. The sensitivity analysis involved randomly splitting the full dataset into a training set (70% of samples) and a validation set (30% of samples) for 10 iterations. In each iteration, both 3-class and 4-class models were fitted to the training set, and their performance was further evaluated on the validation set. Key metrics, including entropy (classification accuracy), VLMRT (significance of fit improvement), and consistency of clinical interpretability, were compared across models. Results confirmed that the 3-class model outperformed the 4-class model in terms of stability, classification accuracy, and clinical relevance:

a. The 3-class model maintained higher average entropy values in both training sets (0.918 vs. 0.902 for the 4-class model) and validation sets (0.905 vs. 0.887 for the 4-class model), indicating more reliable subgroup classification.
b. The VLMRT P-values of the 3-class model remained highly significant across training sets (< 0.001) and validation sets (< 0.001), whereas the 4-class model showed weaker significance (training set: 0.008; validation set: 0.012), suggesting less statistical support for adding a fourth class.
c. The 3-class model exhibited consistent subgroup patterns (low-decline, moderate-ascending, high-ascending-stable) across training and validation sets, with subgroup proportion differences < 5%. In contrast, the 4-class model had unstable proportions of the fourth subgroup (fluctuation > 10% between sets) and a smallest subgroup proportion < 15%, leading to limited clinical utility.

These findings confirm the robustness of selecting the 3-class model.

Page 9, line 205-209:

Based on your suggestion, we have added the result description of sensitivity analysis in “3.2. Identifying exercise self-efficacy trajectories” in the results section, to read “To validate the reliability of the three-class trajectory model, a 10-fold cross-validation was performed as the sensitivity analysis. The full dataset was randomly split into a training set (70% of samples) and a validation set (30% of samples) for 10 independent iterations (S3 Table). These findings indicate that the three-class model provided the best fitting results and was more accurate than the other models.”

Thank you for your thoughtful recommendation.

**Comment 5:**

*Predictor analysis: Were potential interactions (e.g., diabetes × anxiety, social support × income) tested? This may provide deeper insight into subgroup heterogeneity.*

**Response 5:**

Thank you for your thoughtful comments.

We acknowledge that we did not test for potential interactions such as diabetes × anxiety and social support × income in the current study. This was primarily due to two key reasons: First, the sample size distribution across subgroups (64 in Category 1, 100 in Category 2, and 133 in Category 3) would compromise the statistical power to detect interactions reliably. Statistical simulations and methodological literature indicate that testing interaction effects typically requires substantially larger sample sizes than those needed for main effect analyses—our current sample structure, while sufficient for examining main effects, lacks the robustness to identify meaningful interactions without an unacceptably high risk of type II errors (failing to detect true effects).Second, the primary focus of this study was to elucidate the main effects of key predictors, which aligned with our initial research objectives.

We fully agree that testing such interactions could provide deeper insights into subgroup heterogeneity, which is a crucial direction for future research. Therefore, we plan to explore these interactions in future research with an expanded sample size (incorporating more diverse clinical sites and patient populations) to ensure adequate statistical power.

Page 23, line 450-454:

In the Limitation section, we have added the following content: “Fourth, we did not explore potential interactions among predictors (e.g., diabetes × anxiety, social support × income). Future research with larger sample sizes is warranted to investigate these interactions, as they may reveal nuanced differences in how predictors influence exercise self-efficacy across different subgroups, thereby informing more tailored intervention strategies.”

Thank you for your helpful suggestions.

**Comment 6:**

*Implications for practice: The intervention suggestions are broad. Can the authors propose more specific, tailored strategies for each trajectory group?*

**Response 6:**

Thanks for your constructive comments. Based on your suggestions, we have added to the discussion section a description of the characteristics, key predictors, and targeted interventions of the three trajectory subgroups of exercise self-efficacy in patients with multivessel coronary artery disease.

Page 15, line 273-274:

a. For the low self-efficacy decline group, we have added “(characterized by low baseline self-efficacy and continuous decline over time; key influencing factors: history of diabetes, high level of anxiety, and low income)”.

Page 15, line 279 to Page 16, line 292:

In addition, we have added the specific strategies, which is “Healthcare professionals should prioritize these individuals, engage with them through structured interviews, and gain a comprehensive understanding of their difficulties and concerns during exercise. Simultaneously, rehabilitation programs should commence with low-intensity, simple exercises. For patients with diabetes, a blood glucose adaptation exercise program should be developed, including a ten-minute walk one hour after a meal, and patients should be reminded to monitor their blood glucose before and after exercise to avoid the risk of hypoglycemia or blood glucose fluctuations. For patients with anxiety, mindful breathing was integrated into the exercise process, and two minutes of deep breathing training was performed for every ten minutes of exercise to help relieve the tension during exercise. For low-income patients, the community was provided with exercise material support, free pedometers, home resistance bands and other basic tools, and a simple paper exercise record form was designed. Family members assisted in recording the daily exercise time or walking distance of patients, and community medical professionals checked and encouraged exercise data through on-site follow-up or telephone communication every week, enabling patients to rebuild their exercise self-efficacy as they progressively achieve success.”

Page 16, line 292-294:

b. For the high-efficacy ascending-stable group, we have added “(characterized by high baseline self-efficacy, rising first and then stable; key influencing factors: high income, good exercise habits, and high social support)”.

Page 16, line 300 to Page 17, line 308:

What’s more, we have added the specific strategies, which is “For these patients, healthcare professionals can provide advanced exercise recommendations and design personalized advanced exercise plans for high-income patients, such as step-by-step goals from private lessons in the gym to outdoor marathon training. A certification system for sports skills will be established for those who have the habit of exercising, and those who complete events such as swimming or cycling will receive certification badges of rehabilitation institutions. A social support exercise community was built, and an exercise supervision group was formed by family members or friends of patients to carry out collective exercise once a week to help them further improve their physical fitness and encourage them to share exercise experience, thereby inspiring other patients to develop a positive rehabilitation mindset.”

Page 17, line 310-311:

c. For the moderate-efficacy continuous increase group, we have added “(characterized by moderate baseline self-efficacy, continued to rise; key influencing factors: stable social support and gradually developing exercise habits)”.

Page 17, line 314-324:

Additionally, we have added the specific strategies, which is “For these patients, healthcare professionals should maintain the existing rehabilitation guidance, develop a monthly assessment system of exercise efficacy based on the dual path of dynamic adjustment and skill development, and automatically adjust the exercise intensity combined with self-efficacy score and exercise endurance test; The exercise form experience class was set up, and a new exercise, such as Baduanjin and rehabilitation exercise, was introduced every month. The exercise companion partner was formed by community volunteers, and the patients in the same group were paired to supervise each other to clock in. At the same time, the intensity and content of exercise were adjusted in real time according to the progress of rehabilitation to ensure the continuous and steady improvement of self-efficacy, ultimately optimizing rehabilitation outcomes.”

Thank you for your careful considerations.
