# Supplementary material for: Heterogeneous trajectories of exercise self-efficacy and its predictors in patients with multivessel coronary artery disease: A longitudinal study
Source: PLoS One. 2026 Feb 27;21(2):e0339591. doi: 10.1371/journal.pone.0339591 (PMC12948052; doi:10.1371/journal.pone.0339591)
Supplement: Attachment — Submitted filename: reviewer PLOS one.docx [file pone.0339591.s006.docx]

Considering the revisions made by the authors, the following additional points need to be addressed.

1. It is recommended to perform a sensitivity analysis for missing data.
2. Please provide a detailed explanation of the data collection method at each time point. Specify whether the questionnaires were administered online, face-to-face, or via telephone, and whether the method differed across measurement time points (T1–T4).
3. A STROBE flowchart should be included. Please add a participant flow diagram following STROBE guidelines, depicting the number of individuals assessed, excluded, enrolled, followed, and analyzed at each stage.
4. Reference numbers 11 and 17 should be placed inside brackets.
